# Comparative analysis of fatty acids, amino acids, and flavor compounds in different skeletal muscles of the Tianzhu white yak

**DOI:** 10.5713/ab.250878

**Published:** 2026-04-02

**Authors:** Xiangyan Wang, Youpeng Qi, Changze Cui, Shaopeng Chen, Zhihao Luo, Meixian Zhang, Yiduo Dai, Yanbin Zhu, Jiang Hu, Bingang Shi, Shaobin Li

**Affiliations:** 1 Gansu Key Laboratory of Herbivorous Animal Biotechnology, College of Animal Science and Technology, Gansu Agricultural University, Lanzhou, China

**Keywords:** Fatty Acid Metabolism, Meat Flavor, Muscle Fiber Type, Tianzhu White Yak

## Abstract

**Objective:**

Although yak meat is valued for its distinctive flavor profile, little is known about the mechanisms underlying flavor variation among different muscle types. This study aimed to systematically investigate the differences in flavor precursors, metabolic enzyme activities, gene expression profiles, and volatile flavor compounds among three muscles- *longissimus thoracis* (LT), *triceps brachii* (TB), and *semimembranosus* (SM) of the Tianzhu white yak, and to elucidate the underlying regulatory mechanisms.

**Methods:**

A comprehensive approach was employed, integrating targeted metabolomics, untargeted metabolomics, flavoromics, real-time quantitative polymerase chain reaction, and histochemical staining to compare the biochemical characteristics of the three muscles.

**Results:**

The results of volatile profiles and sensory evaluation demonstrated that the TB muscle exhibited more favorable flavor-related traits. The TB muscle was enriched in key flavor precursors, including glutamic acid, glutamine, arginine, and total free amino acid content (p<0.05). Concurrently, the expression levels of key lipogenic genes (*FASN*, *ELOVL4*, *SREBP1*) were significantly higher in the TB muscle (p<0.05). Flavoromics analysis further revealed that the TB muscle exhibited greater diversity and higher relative abundance of key volatile compounds, such as aldehydes, ketones, and alcohols (p<0.05). Sensory evaluation further indicated that the TB muscle exhibited stronger sweet and fruity flavors. Untargeted metabolomics and pathway enrichment analysis indicated that this profile was associated with enrichment of arginine and proline metabolism and unsaturated fatty acid biosynthesis.

**Conclusion:**

This study demonstrates that flavor enhancement in yak meat is associated with the coordinated upregulation of lipid synthesis genes, thereby promoting the accumulation of flavor precursors. This process provides sufficient substrates for the Maillard reaction and lipid oxidation, facilitating the generation of volatile flavor compounds. These findings provide a theoretical basis for yak meat quality evaluation and precision processing.

## INTRODUCTION

The Tianzhu white yak, often referred to as the “white pearl of the grassland”, is an indigenous breed native to the Qinghai-Tibetan Plateau [1]. Unlike conventional cattle species, yaks have adapted to survive under the region’s harsh environmental conditions of the plateau and are typically raised in regions with minimal industrial pollution [2]. For local herders, yaks constitute a vital source of livelihood, providing meat, milk, and other essential resources. Importantly, yak-derived products are widely regarded as natural, pollution-free, and environmentally sustainable. Yak meat is valued by consumers for its distinctive flavor, favorable amino acid profile, and high levels of essential minerals [3]. The livestock meat quality is primarily evaluated by its pH, intramuscular fat content, tenderness, color, flavor, and nutritional value, with flavor being a critical determinant of consumer purchase intention [4]. The formation of flavor compounds is influenced by a range of intrinsic and extrinsic factors, including amino acids and fatty acids profiles, intramuscular fat deposition, muscle fiber type, age, sex, and genetic background [5,6].

Muscle fiber type is considered a key intrinsic determinant of meat flavor. It may regulate flavor development in two main ways: first, by influencing intramuscular fat deposition, which contributes to flavor formation; second, through determining mitochondrial abundance across fiber types, which in turn influences phospholipid content and associated flavor formation. Skeletal muscle fibers are commonly classified into type I and type II based on their c metabolic properties: type I and type II, type I fibers are often referred to as red muscle, whereas type II fibers are termed white fibers [7]. Moreover, these myofiber types can be subdivided into four metabolic categories (slow oxidative, fast glycolytic, intermediate, and fast glycolytic) [8]. Slowly oxidative fibers contain a high mitochondrial density but lower glycogen content and glycolytic enzyme activity, resulting in metabolic differences that markedly influence meat quality [3]. Previous studies have reported that red fibers contain higher myoglobin levels, which are associated with improved tenderness and flavor, whereas white fibers with lower myoglobin content tend to yield meat with a coarser texture [9]. Different muscles in yaks vary in fiber-type composition [10], which likely contributes to the observed differences in flavor profiles. For instance, the LT and TB have been reported to exhibit a weaker beef flavor compared to the *supraspinatus* muscles, 100 whereas, the TB may present a stronger beef flavor than the LT [11]. Recent transcriptomic evidence has further shown that different meat cuts exhibit distinct gene expression patterns, suggesting that muscle-specific molecular regulation may underlie differences in meat quality traits and flavor potential across anatomical locations [12].

The differences in the abundance of flavor precursors are primarily attributed to genetic and metabolic factors, with metabolic enzymes in these pathways playing a key role in regulating flavor formation. The activities of endogenous enzymes involved in lipid and amino acid related pathways regulate the release and accumulation of key precursor pools (free fatty acids, free amino acids), thereby influencing the availability of reactive substrates for subsequent thermal reactions [13]. Recent studies have also highlighted that physicochemical changes and flavor development in animal-derived tissues are closely associated with the transformation of lipid- and amino acid-derived precursors during processing [14]. However, relatively few studies have systematically investigated the metabolites involved in flavor formation in different yak muscles. Therefore, this study aimed to compare muscle fiber characteristics and lipid/amino acid profiles among muscles and to explore underlying mechanisms using metabolomics.

## MATERIALS AND METHODS

### Sample collection

The yaks were obtained from the same herd in Tianzhu Tibetan Autonomous County, Gansu Province, China, and five 4.5-year-old Tianzhu white yaks (males; n = 5) with similar weight (217.50±17.08) and good health status were selected as experimental animals with the consent of the herders. Animals were clinically examined to ensure the absence of disease, injury, or abnormal growth. All yaks had ad libitum access to forage from the same pasture-based feeding system and free access to water. Prior to slaughter, animals were fasted for 24 h with free access to water. The yaks were transported to “Huarui Star Slaughterhouse” a commercial abattoir located in Tianzhu Tibetan Autonomous County, Gansu Province, China, with an approximate transportation duration of 30 min. Slaughter procedures were conducted by professional staff in accordance with standard commercial practices. Yaks were slaughtered in commercial abattoirs by professional slaughterhouse staff.

The LT, TB, and SM were collected, the LT, predominantly composed of slow-twitch fibers, TB and SM contain more fast twitch fibers, specialized for rapid) rinsed with PBS, and rapidly frozen in liquid nitrogen for the determination of flavor compounds, metabolites, amino acids, fatty acids, and gene expression. Additional LT, TB, and SM tissue (2×2×2 cm) was fixed in 4% paraformaldehyde.

### Oil red O staining

The method for Oil red O staining follows the procedure described by Wang et al [15], with specific steps as follows: the tissue was embedded in OCT Reagent (Servicebio), rapidly frozen in liquid nitrogen for 15 s, and frozen in −70°C for 30 min, subsequently thawed in −40°C for 30 min, followed by −20°C for 30 min, and then sectioned into two 10 μm slices. Subsequently, the tissue pieces were fixed on anti-dehiscence slides. For Oil red O staining, sections were rinsed with distilled water for 2 min, immersed in 60% isopropanol for 2 s, stained with oil red O working solution for 15 min, briefly differentiated in 60% isopropanol, rinsed with tap water for 2 min, counterstained with hematoxylin for 30 s, and sealed with neutral resin.

### Metabolic enzyme activity

10% homogenate preparation: weigh 1.0 g of muscle tissue, add 9 mL of pre-cooled saline at the ratio of mass (g) volume (mL) = 1:9, homogenize under the condition of ice-water bath, and centrifuge for 15 min at 3,500 r/min under the condition of 4°C, and then aspirate the supernatant for the assay. The enzyme activity indexes mainly included lactate dehydrogenase (LDH), lactate content (LD) and succinate dehydrogenase (SDH) activity, and ATP were detected using VarioskanLUX multifunctional enzyme labeling instrument (Thermo Fisher Scientific). The steps were performed in strict accordance with the instructions of the kit (Jiangsu Enzyme Labeling Biotechnology).

### Fatty acid composition

The method for fatty acids extraction was adapted from Wang et al [16], with the specific steps outlined below:1 g of the ground sample was weighed, and 0.7 mL of 10 mol·L^−1^ KOH solution and with 5.3 mL of anhydrous methanol were added. The mixture was incubated in a water bath at 55°C for 1.5 h, followed by addition of 0.58 mL of H_2_SO_4_ solution and further incubated at 55°C for 1.5h. After the reaction, the mixture was cooled to room temperature with tap water, followed by the addition of 3 mL of hexane. The supernatant was collected and filtered through an organic phase membrane into a sample vial. Gas chromatography (GC-2010 Plus; Shimadzu) was performed using an HP-88 fused silica capillary column (100 m×0.25 mm×0.20 μm; Agilent Technologies) and a flame ionization detector (FID). Gas chromatography was conducted with nitrogen as the carrier gas, at a flow rate of 450 mL·min^−1^. The injector temperature was set at 260°C; the detector temperature was set 250°C. The oven was initial held at 140°C for 5 min, then increased to 240°C at 4°C·min^−1^ and held for 15 min, with a total run time of 45 min. Identification and quantification were performed using the external standard method with the application of a 37-component fatty acid methyl esters (FAMEs) mixture standard (CRM47885; Supelco; Sigma-Aldrich).

### Determination of amino acid content

Approximately 1 g of yak muscle sample was placed into a centrifuge tube, followed by the addition of 600 μL of methanol formate (Thermo Fisher Scientific). The mixture was centrifuged at 12,000 ×g for 5 min, after with the supernatant was diluted 50-fold with methanol formate and shaken for 30 s. Then, 100 μL of the diluted supernatant was spiked with 100 μL of the Trp-d3 internal standard (100 ng/mL) (Nanjing Reagent), filtered through a 0.22 μm membrane and analyzed.

The LC analysis was performed on EXion LC Liquid chromatography (AB SCIEX). Mass spectrometric detection of metabolites was performed on AB6500 Plus (AB SCIEX). Chromatographic separation was performed on an ExionLC system (AB SCIEX) coupled to a SCIEX QTRAP 6500+ mass spectrometer (AB SCIEX). ACQUITY UPLC BEH C18 Column (2.1×100 mm, 1.7 μm; Waters), the injection volume was 5 μL, the column temperature was set at 40°C, and the mobile phase were A:50 % methanol in water (containing 0.1% formic acid) (Thermo Fisher Scientific) and B:10% methanol in water (containing 0.1% formic acid) (Tokyo Chemical Industry). The ionization was performed using electrospray ionization (ESI) in positive mode. The ion source temperature was set at 500°C, the ion source voltage was 5,500 V, the collision gas was pressure at 6 psi, the curtain gas pressure at 30 psi, and both the atomizing and auxiliary gases at 50 psi. Scans were performed using multiple reaction monitoring (MRM).

### Extraction and detection of flavor compounds

A 500 mg meat sample was placed in a 15 mL centrifuge tube, frozen in liquid nitrogen for 5 min, and homogenized using a pulverizer for 3 min. Then, 0.6 g of the homogenized sample was transferred into a headspace vial containing 10 μL of internal standard solution of n-Hexyl-d13 (C/D/N Isotopes), incubated at 80°C for 10 min, and conditioned at 270°C for 10 min. The conditioned SPME fiber was inserted into the incubation chamber and exposed to the sample headspace for 40 min at 80°C. After adsorption, the fiber was transferred to the GC injection port and desorbed at 250°C for 5 min. For retention index calibration, 10 μL of n-alkanes standard solution (Sigma-Aldrich) was placed in a 20 mL headspace vial. Analyses were carried out using a LECO Pegasus 4D instrument (LECO) consisting of an Agilent 8890A GC (Agilent Technologies) system equipped with a split/splitless injector, and dual stage cryogenic modulator (LECO) coupled with TOFMS detector (LECO). The separation system consisted of a one-dimensional column:Separation was achieved using a DB-Heavy Wax column (Agilent Technologies) for the first dimension and an Rxi-5Sil MS (Restek) for the second dimension. High-purity helium was used as the carrier gas at a constant flow of 1.0 mL·min^−1^. The temperature program of the oven was as followed: the oven temperature was held at 50°C for 2 min at first, then raised to 220°C at the rate of 4°C/min and held for 13 min. The secondary oven temperature was operated at 5°C higher than the first oven. The temperature of the modulator is always 15°C higher than that of the second column. The injector temperature was set to 250°C. Mass spectrometry detection was performed using a LECO Pegasus BT 4D system (LECO) with a transfer line temperature of 250°C, ion source temperature of 250°C, acquisition rate of 200 spectra·s^−1^, electron ionization energy of 70 eV, and detector voltage of 2,031 V.

To compare flavor components with contents across different orders of magnitude, the raw data were normalized using either total peak area normalization or internal standard normalization. VOCs were identified based on the NIST2020 database through annotation with Chroma TOF software. The classification and analysis of detected flavor compounds, including the evaluation of their quantity and relative content, were conducted using the PubChem database and ClassyFire software. Additionally, the sensory characteristics of these compounds were examined and compared using the FlavorDB and Order database. Statistical significance was determined based on variable influence in VOCS (VIP) values>1 and p<0.05.

### Metabolite extraction and analysis

Metabolites were extracted according to the method of Want et al [17]. The procedure was as follows: 100 mg of yak meat from different anatomical regions was weighed and placed into a centrifuge tube containing 1 mL of 70% methanol (Sigma-Aldrich) with internal standard. Samples were homogenized e grinder at 50 Hz for 60 s, and repeated twice. The homogenate was then sonicated at room temperature for 30 min, followed by incubation on ice for 30 min. Samples were centrifuged at 12,000 ×g, and 4°C for 10 min. The supernatant was collected, concentration and dried. Dried exacts were reconstituted in 200 μL of 50% acetonitrile containing 2-chloro-L-phenylalanine (Aladdin) as internal standard, filtered.

The LC analysis was performed on a Vanquish UHPLC System (Thermo Fisher Scientific). Chromatography was carried out with an ACQUITY UPLC HSS T3 (2.1×100 mm, 1.8 μm) (Waters). The column maintained at 40°C. The flow rate and injection volume were set at 0.3 mL/min and 2 μL, respectively. For LC-ESI (+)-MS analysis, the mobile phases consisted of (B2) 0.1% formic acid in acetonitrile (v/v) and (A2) 0.1% formic acid in water (v/v). Separation was conducted under the following gradient: 0–1 min, 8% B2; 1–8 min, 8%–98% B2; 8–10 min, 98% B2; 10–10.1 min, 98%–8% B2; 10.1–12 min, 8% B2. For LC-ESI (−)-MS analysis, the analytes was carried out with (B3) acetonitrile and (A3) ammonium formate (5 mM). Separation was conducted under the following gradient: 0–1 min, 8% B3; 1–8 min, 8%–98% B3; 8–10 min, 98% B3; 10–10.1 min, 98%–8% B3; 10.1–12 min, 8% B3.

Mass spectrometric detection of metabolites was performed on Orbitrap Exploris120 (Thermo Fisher Scientific) with ESI ion source. Simultaneous MS1 and MS/MS (Full MS-ddMS2 mode, data-dependent MS/MS) acquisition was used. The parameters were as follows: dependent MS/MS acquisition was used. The parameters were as follows: sheath gas pressure, 40 arb; aux gas flow, 10 arb; spray voltage, 3.50 kV and −2.50 kV for ESI (+) and ESI (−), respectively; capillary temperature, 325°C; MS1 range, m/z 100–1,000; MS1 resolving power, 60,000 FWHM; number of data dependant scans per cycle, 4; MS/MS resolving power, 15,000. Dynamic exclusion was employed to eliminate redundant MS/MS information.

Relative metabolite abundances were normalized and orthogonal partial least squares-discriminant analysis (OPLS-DA) using R software. Differential metabolites were identified based on variable importance in projection (VIP>1) and p<0.05. Functional pathway enrichment analysis of the filtered differentially expressed metabolites was performed using the Metaboanalyst software.

### Reverse transcription quantitative polymerase chain reaction

Approximately 100 mg of muscle tissue were ground into 1 mL of AG RNAex Pro reagent (Accurate Biotechnology) using a tissue grinder, and subsequent were performed were carried out according to the manufacturer’ instructions (Accurate Biotechnology). The cDNA was synthesized from the extracted RNA according to the reverse transcription kit. Primer5.0 software was used to design the gene primers, and the primer information is shown in Table 1. Relative gene expression was calculated using the 2^−ΔΔCT^ method.

### Statistical analysis

Reverse transcription quantitative polymerase chain reaction (RT-qPCR) data, Oil red O area, amino acids and fatty acids content were statistically analyzed using SPSS 26.0 one-way ANOVA and Tukey post-hoc test. The results have been presented as mean±standard deviation, with statistical significance determined at p<0.05.

## RESULTS

### Analysis of intramuscular fat deposition of different fiber types

**Figure 1:**
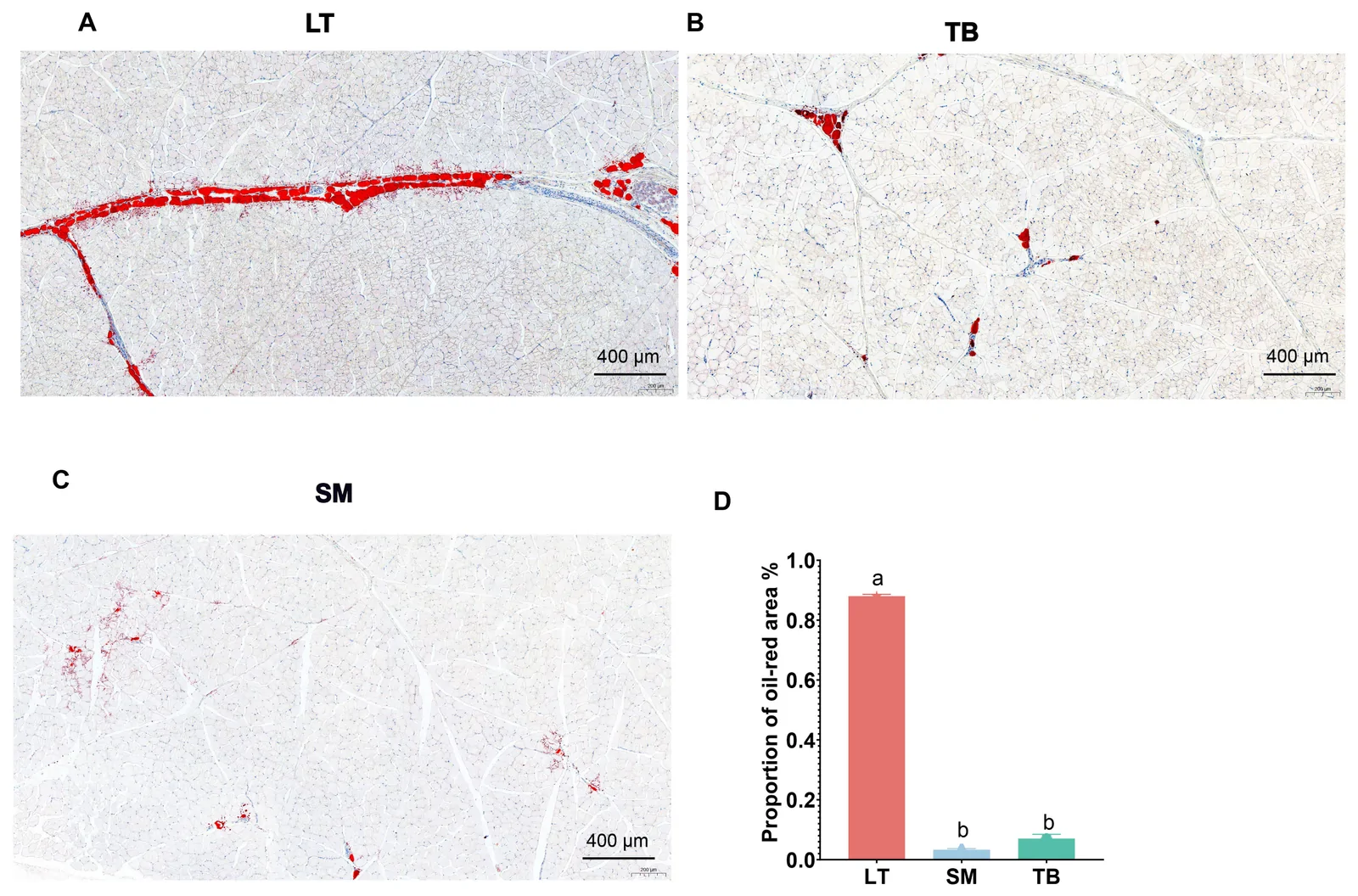
Oil red O staining of different muscle parts of the Tianzhu white yak. Red indicates lipid droplets. (A) LT Oil red O staining image; (B) TB Oil red O staining image; (C) SM Oil red O staining image. Scale bar: 400 μm. (D) Statistical chart of Oil red O staining results for muscle tissues from different regions. ^a,b^ Different lowercase letters a–c above the bars indicates significant differences. LT, *longissimus thoracis muscle*; TB, *triceps brachii*; SM, *semimembranosus muscle*.

As shown in Figure 1, intramuscular fat in the three muscles was mainly distributed as lipid droplets within the inter-fascicular spaces between muscle fiber bundles. Lipid signals were quantified by image analysis based on Oil Red O–stained sections. The results showed that the lipid droplet area fraction in LT was significantly higher than that in SM and TB (p<0.05). In addition, the difference between SM and TB was not significant (p>0.05). The higher intramuscular fat deposition in LT suggests a greater reserve of lipid-derived precursors.

### Muscle metabolizing enzyme activities in different muscle fibers of yak

**Figure 2:**
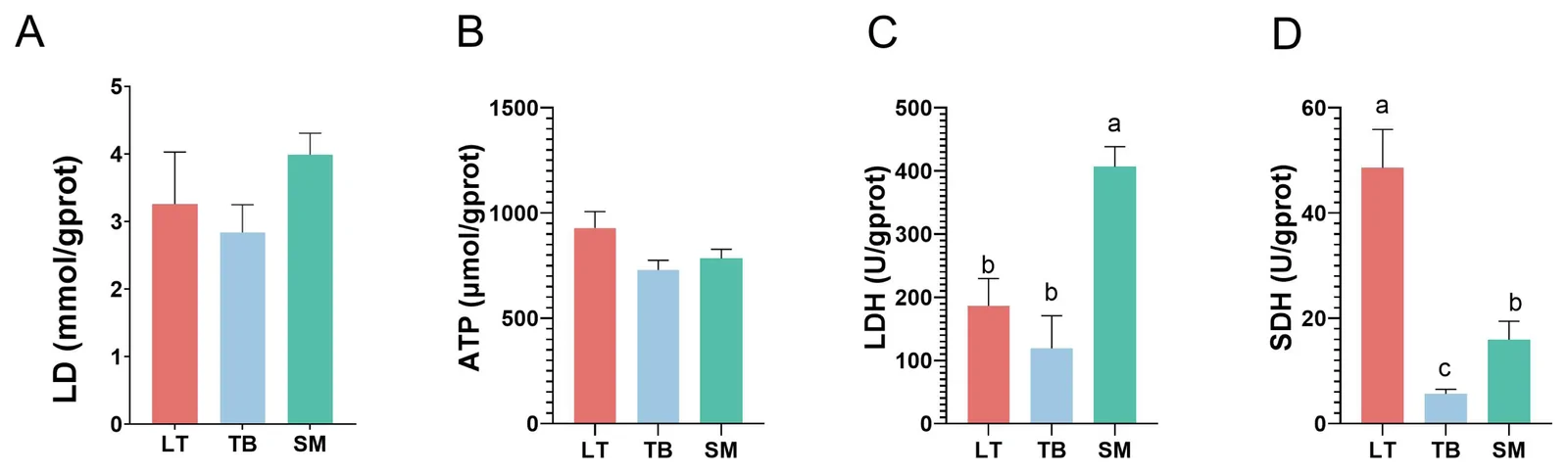
Metabolic enzyme activities in different parts of the muscle. (A–D) indicates Lacte, ATP, Lactate dehydrogense and succinate dehydrogenase activities. ^a–c^ Different letters above the bar charts indicate significant differences between groups (p<0.05). LD, lactate; LT, *longissimus thoracis*; TB, *triceps brachii*; SM, *semimembranosus muscle*; LDH, *lactate dehydrogenase*; SDH, succinate dehydrogenase.

Figure 2 demonstrates significant differences in enzymatic activity among the three muscle types. LDH activity was markedly higher in the SM group compared to TB and LT (p<0.05). In contrast, SDH activity was significantly higher in the LT group relative to the other two muscle types (p<0.05).

### Evaluation of fatty acid indicators in muscle fibers of different types

As shown in Table 2, a total of 29 fatty acids were identified in the three parts of yak, included 13 saturated fatty acids, 8 monounsaturated fatty acids, and 8 polyunsaturated fatty acids. In yak muscle, 18:1n9c and C18:0 was identified as the predominant fatty acids. A total of 12 fatty acids exhibited significant differences among different parts of the muscle, with C18:0, C18:1n9c, C18:2n6c, and C18:2n6t were the highest in TB (p<0.05). Conversely, C14:0, C16:0, C21:0, C23:0, and C18:3n6 were significantly higher in SM compared to the other two groups (p<0.05), while C14:1 and C20:1 were higher in the LT group than in the SM and TB (p<0.05).

### Evaluation of amino acid indicators in muscle fibers of different types

Compared to yak TB, the concentrations of Orn, Gln, Glu, Arg, flavor amino acid (FAA), bitter amino acid (BAA), and non-essential amino acids (NEAA) in LT and SM were lower (p<0.05). In contrast, Hcy, Tyr, Ser, and His were higher in LT than in TB (p<0.05), while the content of NEAA was higher in TB than in LT (p>0.05), and the total free amino acid content was significantly higher in TB than in LT (p<0.05) (Table 3).

### Gene expression of lipid metabolism in different muscle fibers

**Figure 3:**
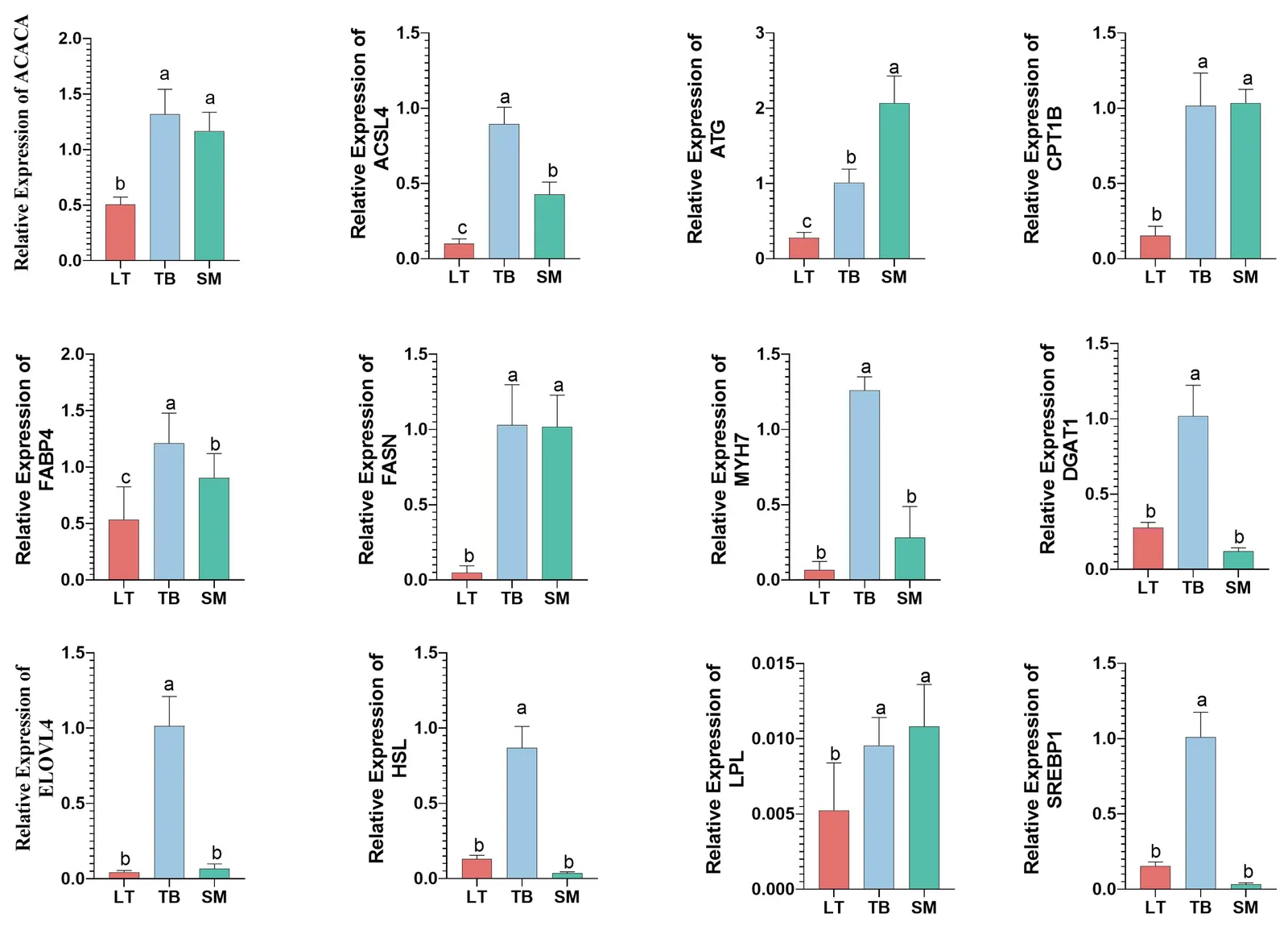
Fatty acid metabolism-related gene expression. ^a–c^ Different letters above the bar charts indicate significant differences between groups (p<0.05). LT, *longest dorsal muscle*; TB, *triceps brachii*; SM, *semimembranosus muscle*.

As shown in Figure 3, the acetyl-CoA carboxylase-α (*ACACA*), carnitine palmitoyltransferase-1b (*CPT1B*), Fatty acid swasynthase (*FASN*), and Lipoprotein lipase (*LPL*) gene expression was significantly higher in the TB and SM groups than in the LT group (p<0.05). The relative expression of myosin heavy chain 7 (*MYH7*), diacylglycerol acyltransferase 1 (*DGAT1*), Elongation of Very Long Chain Fatty Acids 4 (*ELOVL4*), Hormone-sensitive lipase (*HSL*), and Sterol regulatory element-binding factor 1 (*SREBP1*) in the TB group was significantly greater than the in LT and SM groups (p<0.05). The relative expression of Acyl-CoA synthetase long-chain family member 4 (*ACSL4*), Fatty acid-binding protein 4 (*FABP4*) in the TB group significantly greater than the LT and SM groups (p<0.05).

### Comparative analysis of flavor compounds in different muscle fiber type

**Figure 4:**
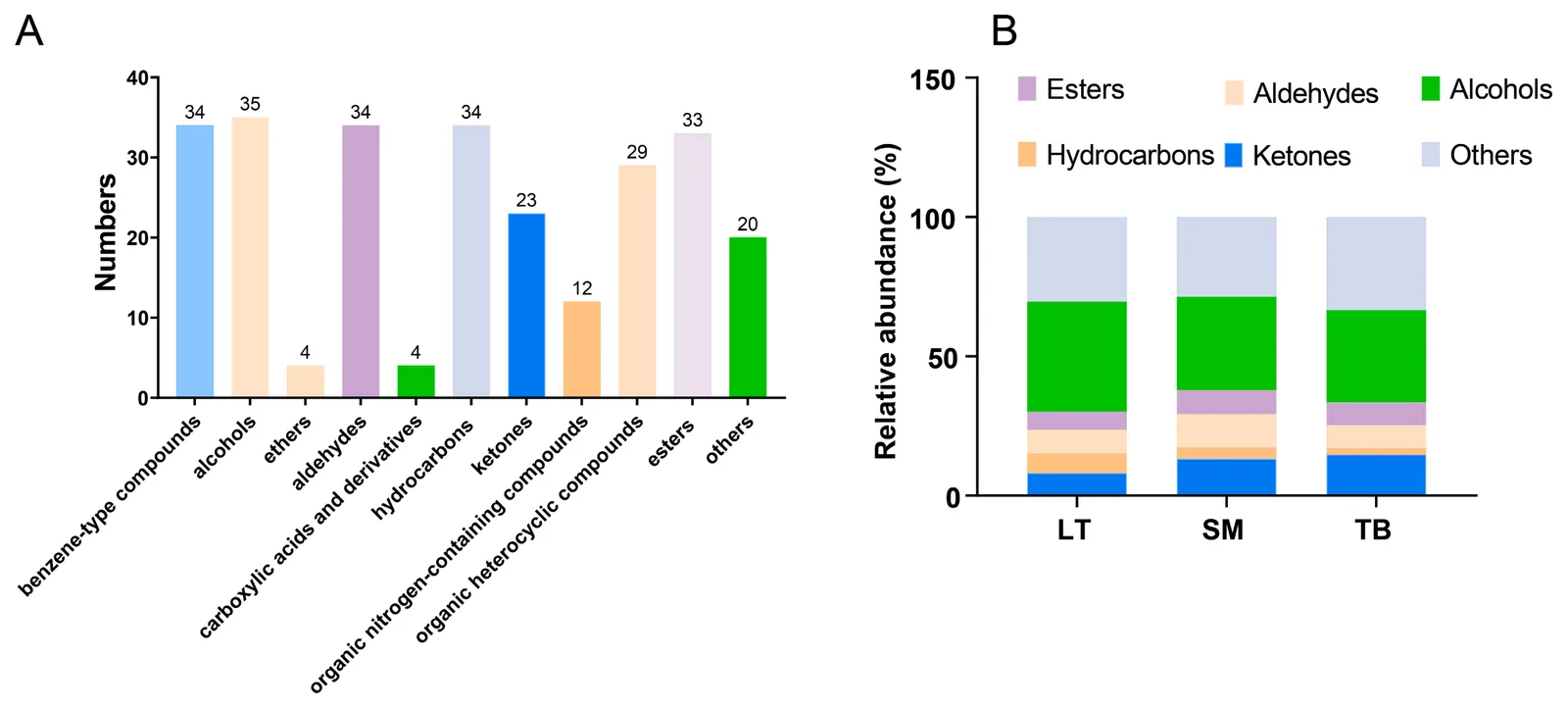
Classification of flavor compounds. (A) Number of each classification of flavor compounds. (B) Relative abundance of flavor compounds classification of three parts of yak meat. LT, *longissimus thoracis*; SM, *semimembranosus muscle*; TB, *triceps brachii*.

A total of 273 flavor compounds were identified after quantifying flavor compounds through a standardized normalization process. These included benzene-type compounds (34), alcohols (35), ethers (4), aldehydes (34), carboxylic acids and derivatives (4), hydrocarbons (34), ketones (23), organic compounds (12), organic heterocyclic compounds (29), lipids and lipid-like molecules (11), esters (33), and others (20) as shown in Figure 4A. The relative content of alcohols in LT was higher than in the other two groups, while the relative content of aldehydes and ketones was lower than in the other two groups. The number of hydrocarbons in SM was higher than in the other two groups, however, the number of heterocyclic compounds, ketones, aldehydes, alcohols, and carboxylic acids in TB was higher than in the other two groups (Figure 4B).

### Analysis of flavor compounds in different parts of yak meat

**Figure 5:**
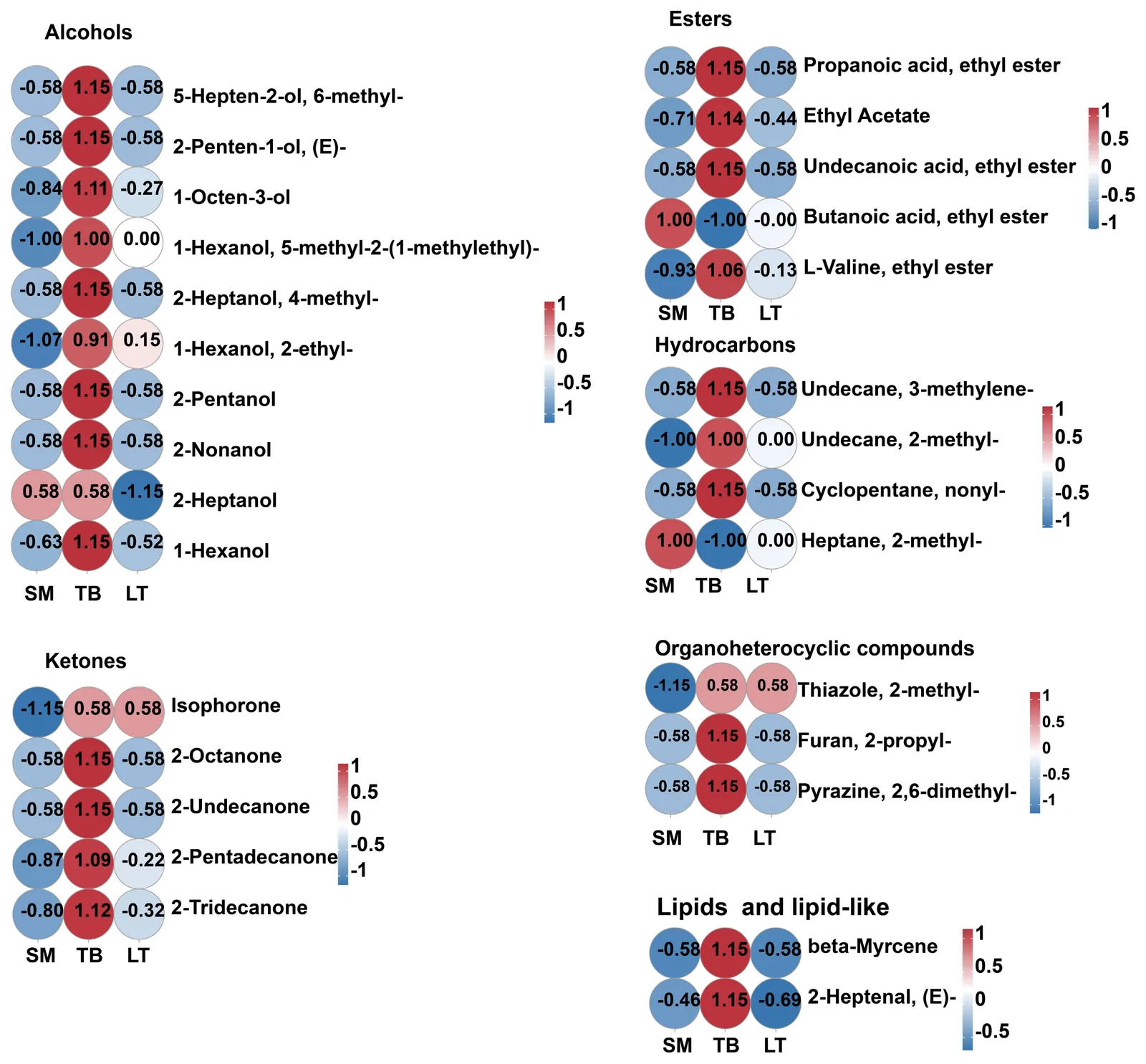
Heat map of differential flavor compounds in different muscle type of the yak. The redder the color indicates a relatively higher abundance of flavor compounds, while the bluer the color indicates a relatively lower abundance of flavor compounds. SM, *semimembranosus muscle*; TB, *triceps brachii*; LT, *longissimus thoracis*.

Through differential analysis, we found that the flavor compounds in the three muscle types were primarily alcohols (Figure 5). Among these, TB contained 5-Hepten-2-ol, 6-methyl-, 2-Penten-1-ol, (E)-, 1-Octen-3-ol, 1-Hexanol, 5-methyl-2-(1-methylethyl), 2-Heptanol, 4-methyl-, 1-Hexanol, 2-ethyl-, 2-Pentanol, 2-Nonanol, and 1-Hexanol were significantly higher in TB than in SM and LT (p<0.05), while the content of 2-Heptanol was significantly higher in LT than in SM and TB (p<0.05). The content of panic acid, ethyl ester, ethyl acetate, undecanoic acid, ethyl esters, and L-valine, ethyl esters in the TB group, was significantly higher than that in the SM and LT groups (p<0.05). For ketone compounds, we found five different flavor compounds, with the content of isophore in SM significantly higher than that in TB and LT (p<0.05). The content of 2-octanone, 2-undecanone, 2-pentadecanone, and 2-tridecanone in TB was significantly higher compared with LT and SM (p<0.05).

### Threshold analysis of flavor compounds

This approach allows for the calculation of the relative odor activity value (ROAV), which assesses the individual impact of volatile compounds on the overall odor. A ROAV value of ≥1 indicates that a compound is a significant contributor to the overall scent. From the Table 4, it can be seen that SM contains 8 key odor compounds, 7 key odor compounds in LT and 6 key odor compounds in TB. The highest flavor compounds in all samples were 2-Nonenal, (E)-, and 2,3-Butanedione.

### Sensory characterization of differential flavor compounds

**Figure 6:**
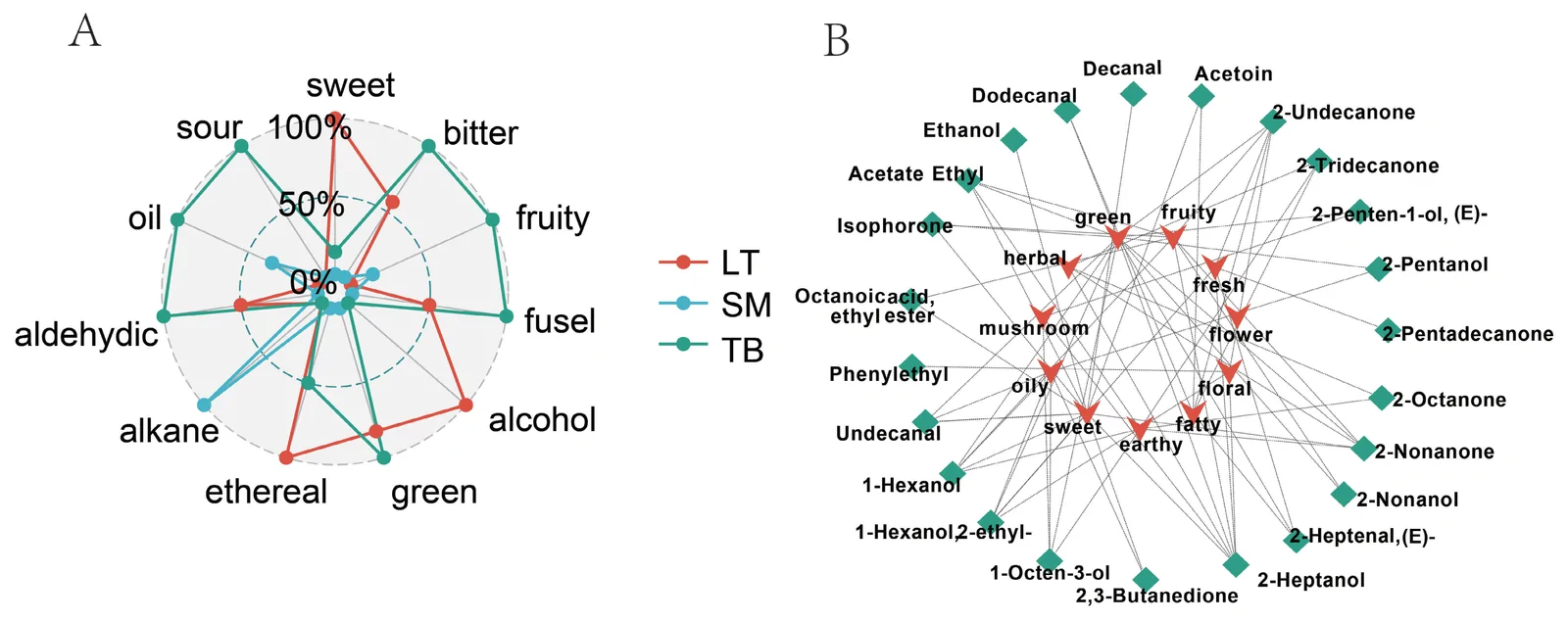
Sensory analysis of flavor compounds. (A) Sensory radar maps of different parts meat of the yak. (B) Network diagram of flavor compounds and sensory characteristic. The inverted triangle indicates flavor sensory characteristics, and the diamond shape indicates volatile organic compounds. LT, *Longissimus thoracis*; SM, *semimembranosus muscle*; TB, *triceps brachii*.

To obtain a comprehensive understanding of the sensory characteristics variation in yak meat from different parts, we conducted a sensory evaluation using the Flavordb and order database. Radar plots of flavor profiles were created, and as depicted in the figure, the sensory evaluation results indicated that yak meat exhibited higher sweetness and green aroma, contributing to a fresh and pleasant odor. Among these, the TB group displayed the strongest sweet and fruity flavors, followed by the LT group, while the SM group had relatively weaker odors. The radar chart illustrates that the TB part of yak provides a general profile of pleasant flavor characteristics (Figure 6A).

We created network maps of flavor compounds and their sensory properties using Cytoscape. We chose three sets of different volatile flavor compounds, with the 10 most common flavors for constructing the network map. 3-heptanal, (E)-, 2-undecanone, ethyl undecanoate, and 2-tridecanone are associated with a fatty flavor. 2-Heptanol, 3-Heptenal, (E)-, 2-Undecanone, 2-Tridecanone, 1-Hexanol, 2-Ethyl- play a crucial role in yak flavor (Figure 6B).

### Metabolomics data analysis

**Figure 7:**
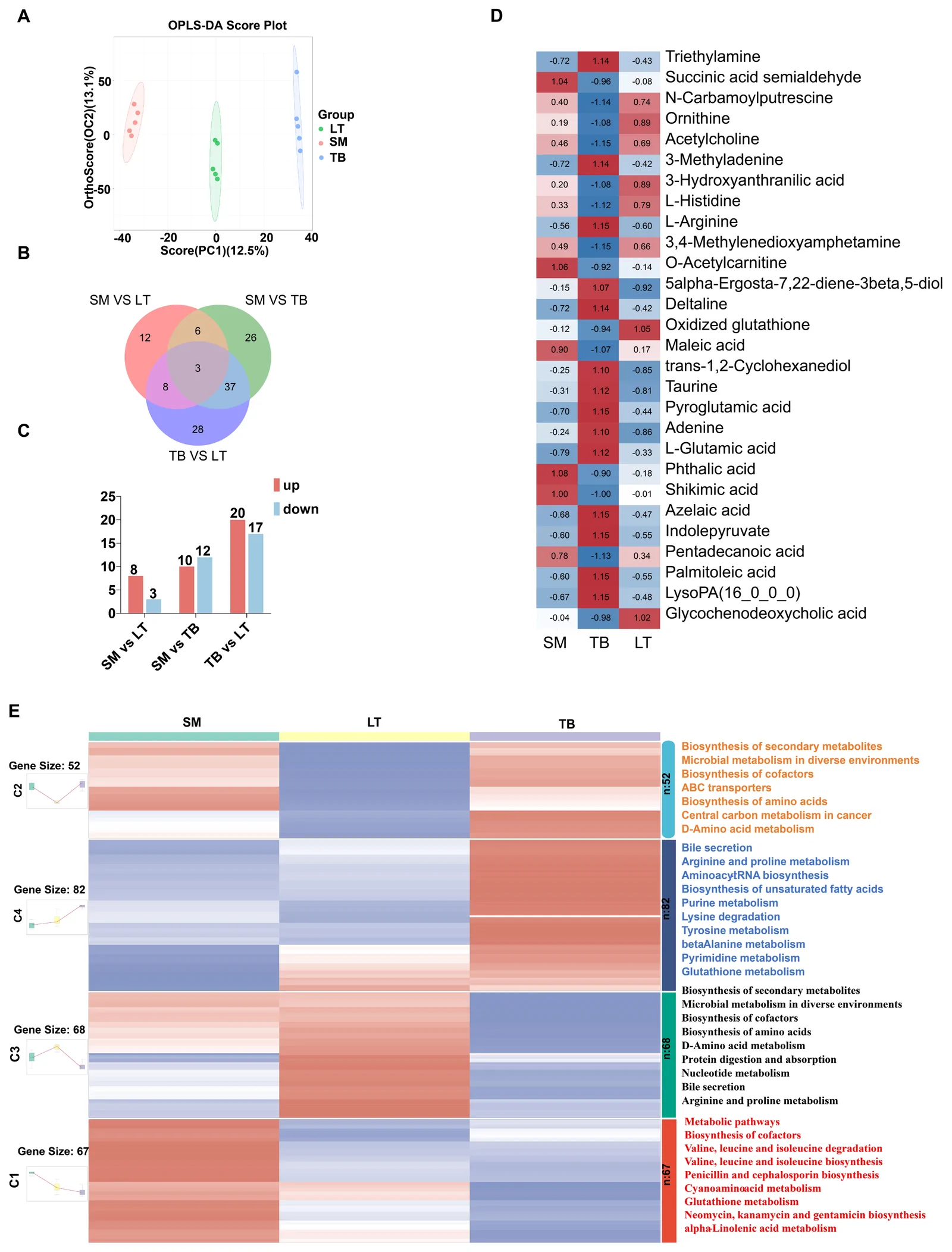
Differential metabolite profiling analysis. (A) OPLS-DA analysis of metabolomic data from different muscle types. (B) Different metabolite Venn plots analysis for different muscle types in yak. (C) Different metabolite histograms analysis for different muscle types in yak. (D) Heatmap analysis of metabolite differences across distinct muscle types in yak. (E) An analysis of differential metabolites using a fuzzy c-means algorithm and enrichment analysis. OPLS-DA, orthogonal partial least squares-discriminant analysis; LT, *Longissimus thoracis*; SM, *semimembranosus muscle*; TB, *triceps brachii*.

Broadly targeted metabolomics analysis revealed 270 metabolites in different parts of all yaks belonging to 12 classes. This suggests that the models generated from OPLS-DA were well-fitted and had a high level of predictability, making them suitable for further analysis of the data. Subsequently, the groups were carefully examined and classified for distinguishing metabolites using the OPLS-DA results (Figure 7A). There are three differentially enriched metabolites common to all three comparison groups (Figure 7B). A total of 11 differential metabolites were identified in SM vs LT (8 upregulated, 3 downregulated). In SM vs TB, 22 differential metabolites were identified (10 upregulated, 12 downregulated), and 37 metabolites were identified in TB vs LT (20 up-regulated, 17 down-regulated) (Figure 7C). In general, comparisons between the three parts identified 28 differential metabolites (Figure 7D).

Subsequently, a trend analysis was performed on 270 metabolites using the fuzzy c-means algorithm, which revealed four main clusters. Additionally, Kyoto Encyclopedia of Genes and Genomes (KEGG) pathway enrichment analysis was conducted for each trend cluster (Figure 7E). Metabolites highly expressed in TB muscle were primarily enriched in pathways related to amino acid, fatty acid, and purine metabolism, all of which are associated with the synthesis of flavor precursor compounds. These metabolites, which are highly abundant in TB muscle, may be one of the primary reasons for the high content of flavor compounds in TB.

**Figure 8:**
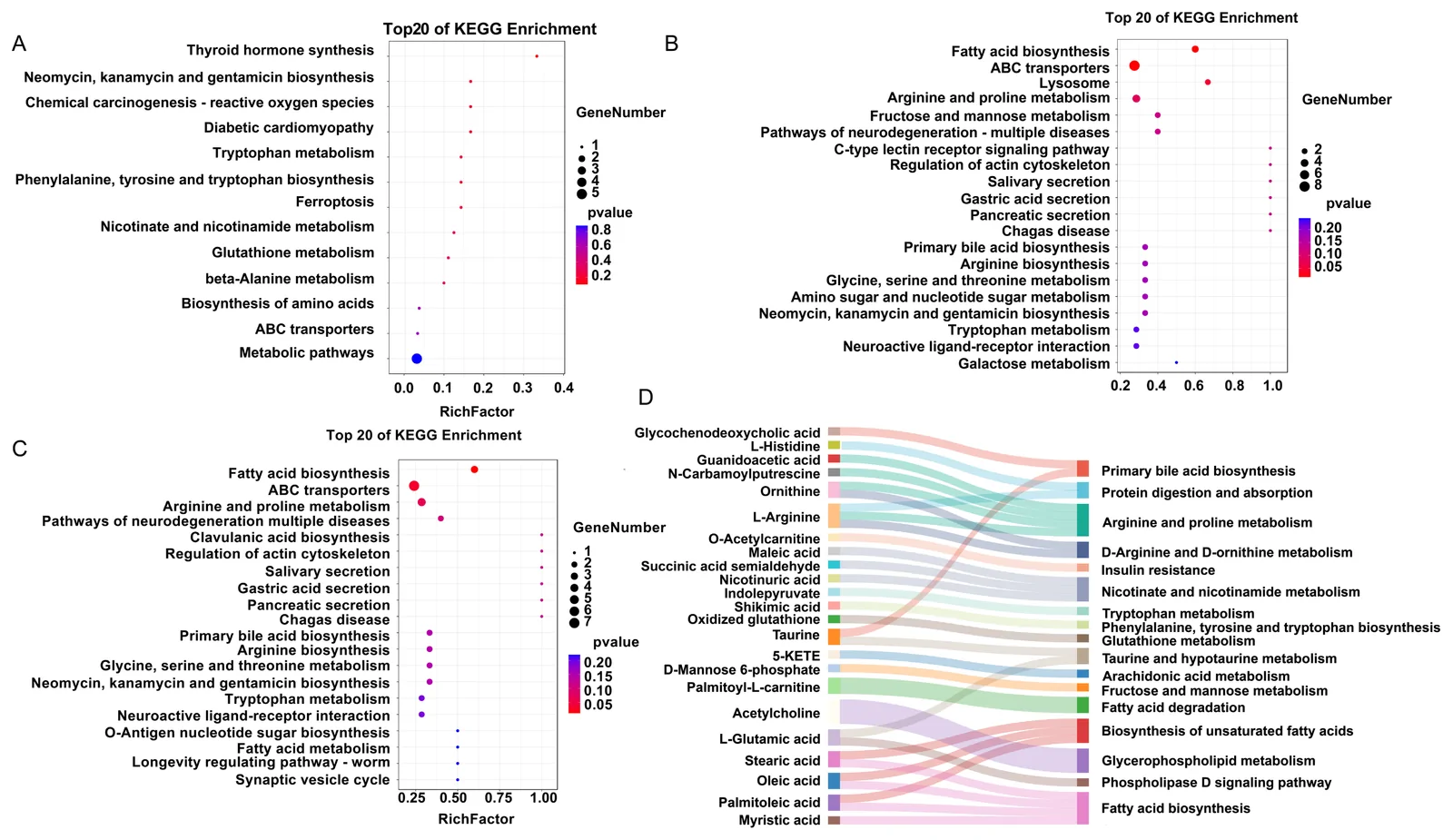
Differential metabolite enrichment analysis. (A) KEGG plots of differential metabolite enrichment in SM vs LT. (B) KEGG plots of differential metabolite enrichment in SM vs TB. (C) KEGG plots of differential metabolite enrichment in TB vs LT. (D) KEGG plots of differential metabolites associated with fatty acid. KEGG, Kyoto Encyclopedia of Genes and Genomes; SM, *semimembranosus muscle*, LT, *Longissimus thoracis*; TB, *triceps brachii*.

Functional enrichment analysis of different metabolites was conducted to identify enriched metabolic pathways in the KEGG pathway. The metabolic pathways of differential metabolites included metabolic pathways, fatty acid biosynthesis, and amino acid metabolism biosynthesis. Enriched metabolic pathways between SM and LT were found to be tyrosine metabolism, phenylalanine, tryptophan metabolism, thyroid hormone synthesis, and Glutathione metabolism (Figure 8A). Additionally, tyrosine, alanine, phosphatase lipid D, and fatty acid biosynthesis were significantly enriched in SM vs TB (Figure 8B), while fatty acid biosynthesis and tyrosine. Synthesis-related pathways were significantly enriched in TB and LT (Figure 8C). The biological processes of these differential metabolites-enriched KEGG pathways indicated their involvement in amino acid, carbohydrate, and lipid metabolism. In addition, considering that flavor compounds are mainly produced with lipid metabolism and amino acid reactions, thus differential metabolites related to fatty acid and amino acid metabolism were identified. Functional enrichment analyses of these precursors that may be useful for flavor revealed that these differential compounds were mainly enriched in the pathways of fatty acid and unsaturated fatty acid and glycerophospholipid metabolism, and therefore we hypothesized that changes in fatty acid content were the main reason for the flavor differences (Figure 8D).

## DISCUSSION

### Intramuscular fat different muscle fibers

Muscle fiber types influence metabolic rate, connective tissue abundance, intramuscular fat deposition, and postmortem proteolysis, thereby affecting meat color, tenderness, texture, flavor, juiciness [18]. Meat quality is primarily reflected through indicators such as muscle tenderness, cooked yield, water loss rate, and intramuscular fat content. Among these, intramuscular fat content plays a decisive role in determining meat tenderness, juiciness, and flavor. Previous studies have demonstrated a positive correlation between intramuscular fat content and meat tenderness [19]. In this study, Oil-red O staining showed that lipid droplets were primarily located in the muscle fibers, and the Oil-red area of LT was significantly greater than that of TB and SM, suggesting higher intramuscular fat content in LT and potentially improved sensory attributes, including tenderness and palatability. The differences in Oil-red O staining among the three muscles may reflect variation in muscle fiber composition and functional demands; compared with the LT, TB and SM are more specialized for locomotion and weight-bearing, which may limit intramuscular fat deposition. Bai et al. reported that the *infraspinatus* muscle displayed a more intense color and higher intramuscular fat content that thefi longissimus thoracis and *semitendinosus* muscles [20]. These findings are consistent with previous studies that muscle fiber type is a key determinant of intramuscular fat distribution, which in turn influences meat tenderness and flavor [21].

### Metabolic enzyme activity of different muscle fiber types

Muscle fiber type composition is a key determinant of muscle metabolic characteristics and plays a critical role in shaping meat quality, particularly flavor development. Lactate dehydrogenase is involved in the glycolytic pathway and catalyzes the production of lactic acid from pyruvate, a hallmark enzyme that measures the body’s capacity for anaerobic fermentation and aerobic metabolism [22,23]. Oxidative muscles typically contain more mitochondria and phospholipids, which can increase the availability of specific PUFAs and lipid-derived flavor precursors. Therefore, the combined differences in intramuscular fat deposition and metabolic enzyme activities likely contribute to muscle-specific precursor availability and oxidative stability. Succinate dehydrogenase activity is positively correlated with the number of oxidized muscle fibers and negatively correlated with the number of enzymatic muscle fibers in muscle [24]. Succinate dehydrogenase is not only involved in meat energy metabolism, but also plays a central role in flavor generation. It affects the formation of color, aroma and “umami” by regulating succinic acid metabolism, electron transport and myoglobin status. In the meat industry, the precise regulation of SDH activity is a potential path to improve flavor quality. For example, Gao et al in the higher class added different concentrations of succinate to the *longissimus thoracis* of beef, and found that higher concentrations of succinate could enhance the myoglobin and flavor of meatloaf [25]. In this study, the content of SDH in the LT group was higher than that in TB and SM, which may be due to structural differences within the muscles. This result suggested that differences in muscle fiber type drive distinct energy metabolic patterns, which in turn influence the activity of key metabolic enzymes like SDH and LDH. Therefore, fiber-type composition indirectly but significantly affects the flavor profile and overall eating quality of yak meat.

### Amino acid content of different muscle fiber types

Free amino acids are crucial flavor precursors, as their types and concentrations directly influence the sensory quality of meat [26]. Notably, Lys, Arg, Asp, and Glu are recognized to flavor compound formation by interactions with soluble reducing sugars in meat [27]. Glu exhibited the highest concentrations and is recognized as a key flavor precursor, capable of synergize with inosinic acid to enhance a range of flavors, including acidic and alkaline flavors [28]. In this study, Glu levels were higher in TB than in LT, suggesting that yak TB meat exhibited a more pronounced flavor profile. Interestingly, total free amino acid content in TB was significantly higher than in LT, which further supports the notion that higher amino acid content in TB may facilitate the generation of more volatile flavor compounds. This could be particularly relevant in the context of the Maillard reaction, where amino acids interact with reducing sugars to form volatile compounds that contribute to the aroma and flavor of the meat. Although this study suggests a correlation between elevated amino acid levels and enhanced flavor profiles in TB, further studies are needed to confirm the specific pathways involved in flavor compound synthesis, particularly those linked to the Maillard reaction.

### Fatty acid content of different muscle fiber types

Fatty acids, amino acids, and proteins are important sources of nutrients in beef cattle, and studies have shown that the composition and content of fatty acids and amino acids have a significant effect on the flavor, juiciness, and texture of meat [29,30]. The impact of fatty acids on hardness is attributed to their varying melting points, which decrease as unsaturation increases [31]. Higher levels of UFAs in lamb lead to quicker fat oxidation and a shorter shelf life [32]. Oleic acid, due to its single double bond and relatively reactive molecular structure, oleic acid readily undergoes oxidative degradation and participates in complex chemical reactions such as the Maillard reaction and Strecker degradation. These processes lead to the formation of key volatile compounds including aldehydes, ketones, esters, and alcohols. These volatiles contribute to the characteristic aroma and flavor profile of cooked meat by providing notes ranging from fatty and buttery to green, fruity, and floral tones [33]. In this study, we found that the oleic acid content in the three groups there were significant differences, which may have contributed to the differences in the flavor of the muscles of different parts of the yak. Similarly, it was also found that the higher oxidative capacity of unsaturated fatty acids, produces a rich flavor profile during meat processing, and we found that the distinctive flavor of yak may be related to the richness of unsaturated fatty acids in the muscle [34]. In the present study, we found that the C16:0 content in SM was significantly higher than the other two groups, which may be beneficial for the synthesis of SM aldehydes. C16:0 is important for flavor formation and can be oxidized to form heptanal, nonanal, 2-heptanone, and D-limonene. Heptanal has a fishy and sweet almond flavor. Nonyl aldehyde gives fats an aromatic, herbaceous, and fishy flavor. 2-heptanone and D-limonene have a fruity flavor [33].

### Fatty acid synthesis gene expression in different muscle fiber types

In this study, we analyzed the expression of genes related to fat deposition in different muscle fiber type of yak. *ACACA* and *FASN*, as key enzymes for fatty acid synthesis, promoted the synthesis of long-chain fatty acids, while *LPL* mediates the uptake of circulating lipids for storage in adipose tissues [35]. *ELOVL4* is responsible for lengthening fatty acid chain lengths, promoting the formation of long-chain and ultra-long-chain fatty acids, which are readily converted into flavor substances [36]. *SREBP1* is a critical transcription factor involved in fat synthesis [37]. Their high expression may promote the bidirectional regulation of lipid metabolism in the TB group, which allows for the co-existence of fat storage and mobilization, thus contributing to the formation of specific fat flavor profiles. There are significant differences in the expression characteristics of genes related to fat metabolism in different parts of yak, and these differences not only affect the fat deposition pattern, but also may regulate the fatty acid composition, accumulation of flavor precursors, and protein degradation pathways, which ultimately shape the flavor characteristics of meat from different parts of yak.

### Flavor compounds in different muscle fiber types

Among all compound classes, aldehydes are identified as the predominant contributors to yak meat aroma due to their low odor thresholds and strong sensory impact [38]. Aldehydes are primarily formed through oxidation of unsaturated fatty acids or the decomposition of lipid-derived radicals [31], with their relative abundance increases with intramuscular fat content, which serves as a substrate for lipid oxidation [39]. At lower concentrations, aldehydes contribute to characteristic fatty aroma, whereas higher concentrations may generate rancid or off-flavors [40]. Gkarane et al reported that the aldehydes are formed through the autoxidation of C18:1 n9 and C18:2 n6, imparting orange, onion, and green odor [41]. In this study, the levels of 2-methylpentanal, 2-methylpropanal, 3-methylbutanal, and 2-methylbutanal were significantly higher in TB than in LT. We hypothesized that the elevated 2-nonenal content in TB is attributable to its higher unsaturated fatty acids content compared to LT, which in turn leads to increasing aldehydes levels by lipid oxidation.

A total of 6 key flavor compounds were identified across different regions of yak meat. Among these, 2,3-Butanedione exhibited the highest ROAV value, indicating that it contributes most significantly to yak meat aroma. This finding is consistent with the report by Yu et al who identified 2-nonenal, (E)-, 2-octenal, heptanal, 1-octen-3-one, 2,3-butanedione, and furan, 2-pentyl- as the six key aroma compounds in Gannan yak across different age groups [42]. The convergence of these results underscores the importance of these 6 compounds in defining the characteristic aroma of yak meat. Furthermore, Wan et al. recognized 2,3-butanedione as a key aroma compound in both raw and cooked beef [43], and a comparative regional study on air-dired beef also highlighted 2,3-butanedione as a key flavor compound [44]. Overall, compared to LT and SM, TB exhibits enhanced flavor characteristics across most aroma attributes. Although the ROAV of 2,3-Butanedione in TB is lower than that in LT and SM, the higher abundance of multiple key flavor compounds in TB collectively shapes its aroma profile through complex cumulative, antagonistic, and synergistic interactions.

### Metabolites in different muscle fiber types

Flavor-associated metabolites differ among muscles, including O-Acetylcarnitine, taurine, -L-carnitine, arginine, and glycylcholic acid. In addition to contributing to nutritional value, taurine binds to bile acids [45], regulates of glucose and lipid homeostasis, membrane stabilization, antioxidant, and metabolic regulatory functions [46]. Bile acids play a critical role in signaling, maintaining cholesterol balance, and regulating glucose and lipid metabolism [47]. Primary bile acids are formed through conjugation with taurine, and these taurine-conjugated primary bile acids are subsequently secreted into bile and enter the intestines, enhancing the absorption of nutrients, lipids, and fat-soluble vitamins [48]. Therefore, taurine and cholic acid metabolites, by participating in the regulation of lipid, glucose and energy metabolism, may play potential bioactive roles in the synthesis and regulation of flavor compounds [49]. Additionally, carnitine plays a potential role in lipid and energy metabolism by facilitating and transport of fatty acid into mitochondria for β-oxidation [50]. Although taurine and carnitine are not directly involved in flavor compounds biosynthesis, they may indirectly influence meat flavor by modulating of lipid metabolism.

**Figure 9:**
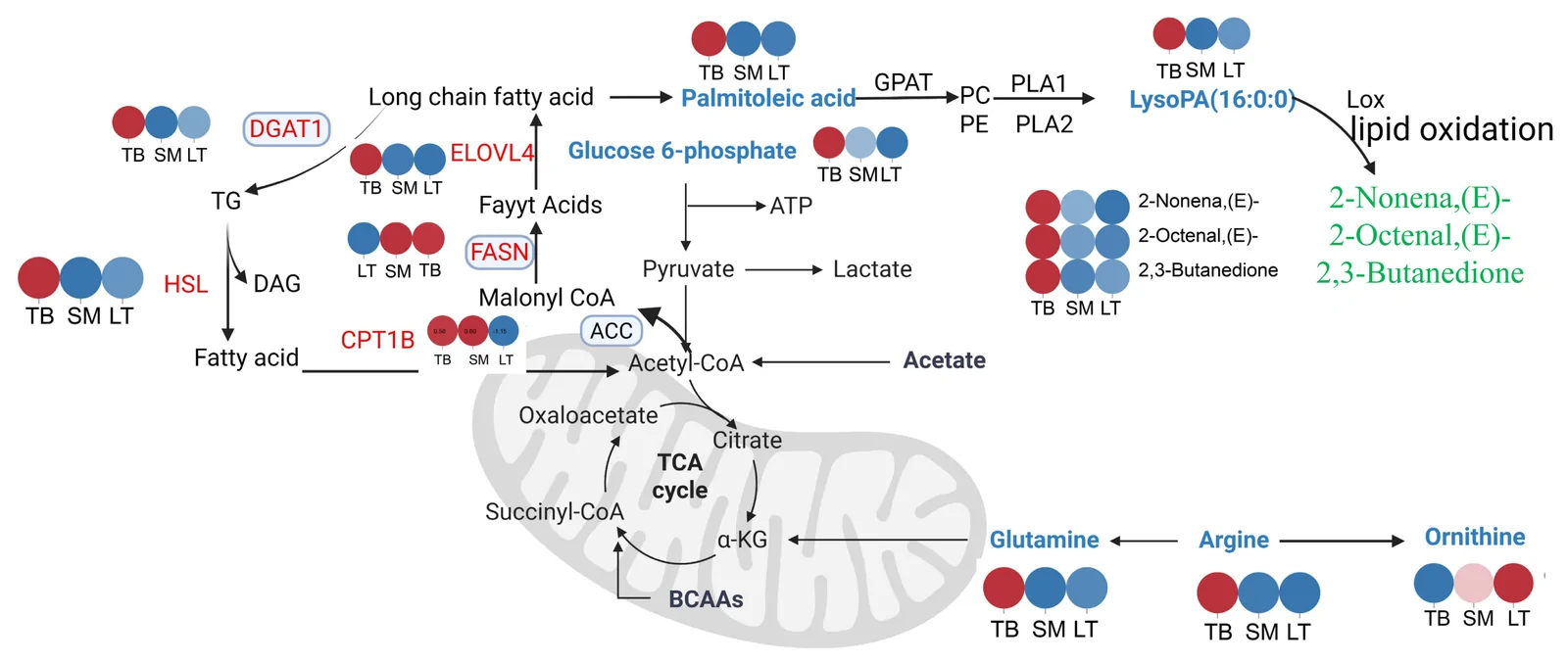
Relationship between flavor compounds and flavor precursors produced in the muscles of three parts of yak. Blue, red, and green fonts indicate compounds that differ between the three sites. Blue fonts indicate metabolites with the highest levels in TB. Red fonts indicate the highest levels of genes in TB. Green fonts indicate the highest levels of volatile organic compounds in TB. TB, *triceps brachii*; SM, *semimembranosus muscle*; LT, *Longissimus thoracis*.

LysoPA (16:0/0:0) (lysophosphatidic acid with a 16:0 acyl chain) is a bioactive lysophospholipid that serves as an intermediate in phospholipid remodeling by the Lands’ cycle and as a signaling molecule modulates cellular responses to oxidative stress [51]. It is typically generated through phospholipase A1 or A2 (PLA1/PLA2) mediated cleavage of phosphatidic acid or other phospholipids [51]. Elevation LysoPA (16:0/0:0) levels may reflect enhanced membrane lipid turnover or stress-induced phospholipid catabolism, processes often accompanied by increased reactive oxygen species [52]. These oxidative conditions favor the peroxidation of polyunsaturated or monounsaturated fatty acids, leading to the formation of volatile aldehydes such as (E)-2-Nonenal and (E)-2-Octenal via β-scission of hydroperoxides formed through lipoxygenase (LOX) or non-enzymatic lipid oxidation pathways [53]. As shown in Figure 9, genes and metabolites associated with fatty acid synthesis are highly expressed in TB. We speculate that the increase in flavor compound content in TB muscle may result from the high expression of genes. This elevated gene expression may promote fatty acids synthesis and accumulation, thereby providing additional substrates or precursors for flavor compounds formation, ultimately enhancing flavor compound content. However, these findings require further validation. Future studies may validate these results by monitoring changes in lipids and flavor compounds through the overexpression and interference of genes involved in fat synthesis in yak adipocytes.

## CONCLUSION

In conclusion, the superior flavor profile of the TB muscle was associated with its enriched levels of key flavor precursors (glutamic acid, glutamine, arginine) and volatile compounds. This enhanced flavor was likely attributable by the upregulated expression of lipid biosynthesis genes, which facilitated the accumulation of flavor-active fatty acids through lipid metabolic pathways. Enrichment analysis of differential metabolites further highlighted the involvement of tyrosine, alanine, and phosphatidic acid metabolism in flavor development. Collectively, this study characterizes the distinct flavor attributes of different yak muscles, providing a scientific basis for optimizing meat quality and enhancing consumer acceptability.

## Figures and Tables

**Table 1:** Primer information for RT-qPCR

Gene	Forward primer	Reverse primer
*ACACA*	GAGGTAGGACGGGTAGGTGT	CAGTCCTCCCTGTAAGAGCC
*ACSL4*	TACATGAACGCCACCAACCA	CATTGAGCTGAGTCGGAGCA
*ATG*	GTGTAGGGGAGCTTGGGGAT	GTCCGCTGACCTATTTGGCA
*CPT1B*	AGCAAGGAGGTGCTCGATTC	CTTCACCAGGGTGCCGTT
*FABP4*	CCTTTGGGGACAGATGCCTT	TACTTGGCTCTTTCAGGGCG
*FASN*	ATTCACAGCCCTCCGGATTC	CTCTGACGGTGCGTTCTCTT
*MYH7*	TCGGGCCTCAACAACATCAA	CTCTTCCCTCCTTAGGGGCT
*DGAT1*	TGATTCTCTCGGTCAGTCAGC	GATGCCACGGTAGTTGCTGAAG
*ELOVL4*	CTTCCCCAACCGCATCCTC	CCCGCCTTGAAGAACACCT
*LPL*	CCATTTTGCCCTGATCCACT	TTAGTTCCACCACTTGCCTC
*SREBP1*	AACATTACTGGCTGGCTGGACAAG	TGGACCGCCCTCTGCTTCAC
*β-actin*	AGCCTTCCTTCCTGGGCATGGA	GGACAGCACCGTGTTGGCGTAGA

RT-qPCR, reverse transcription quantitative polymerase chain reaction.

**Table 2:** Comparison of the relative fatty acid contents of three parts of yak muscle (%)

Fatty acid (%)	TB	SM	LT	p-value
C6:0	0.17±0.04	0.24±0.01	0.17±0.01	0.356
C10:0	0.22±0.06	0.14±0.04	0.16±0.06	0.686
C8:0	0.09±0.03	0.19±0.02	0.11±0.02	0.108
C12:0	0.22±0.04	0.16±0.05	0.16±0.05	0.595
C13:0	0.18±0.04	0.28±0.03	0.27±0.01	0.305
C14:0	1.29±0.45^b^	5.28±0.48^a^	3.46±0.32^ab^	0.045
C15:0	0.41±0.08	0.75±0.26	0.75±0.26	0.517
C16:0	9.33±3.15^b^	11.14±3.15^a^	5.65±1.55^c^	0.046
C17:0	1.39±0.10	1.04±0.06	2.21±0.43	0.556
C18:0	26.20±1.41^a^	18.56±0.86^b^	20.21±2.98^b^	0.033
C20:0	0.40±0.05	0.30±0.01	0.42±0.06	0.432
C21:0	12.58±0.75^b^	15.4±0.38^a^	9.21±0.04^c^	0.000
C23:0	3.67±0.63^b^	5.81±0.62^a^	5.37±0.59^a^	0.045
C14:1	2.29±0.66^b^	0.82±0.32^c^	4.19±1.11^a^	0.041
C17:1	0.38±0.08	0.86±0.39	0.42±0.13	0.378
C16:1	3.41±0.65	4.97±0.28	3.69±0.49	0.092
C18:1n9t	0.52±0.08^b^	1.19±0.37^a^	0.56±0.15^b^	0.036
C20:1	5.32±0.54^a^	2.51±0.04^b^	6.3±0.81^a^	0.006
C15:1	1.45±1.05^a^	0.30±0.06^c^	0.85±0.25^b^	0.012
C24:1n9c	0.81±0.07^b^	1.43±0.46^a^	1.8±0.84^a^	0.002
C18:1n9c	27.42±2.3^a^	21.09±0.59^b^	23.20±2.81^b^	0.033
C18:2n6c	1.48±0.30^a^	0.22±0.01^c^	0.97±0.06^b^	0.000
C18:3n6	0.53±0.21^b^	2.39±1.02^a^	1.47±0.75^ab^	0.036
C18:2n6t	3.28±0.72^a^	2.45±0.60^b^	1.98±0.26^b^	0.047
C18:3n3	0.76±0.37^ab^	0.35±0.05^b^	1.31±0.78^a^	0.041
C20:3n3	0.54±0.04	0.54±0.04	0.73±0.09	0.401
C20:4n6	5.93±1.23	3.5±1.87	4.5±2.07	0.645
C20:5n3	3.82±1.64	3.42±0.58	3.92±1.82	0.631
C22:6n3	1.6±0.92^b^	1.13±0.32^b^	3.52±0.21^a^	0.000

a–c indicate those with different subscripts in the same row, significant difference at p<0.05.

TB, *triceps brachii*; SM, *semimembranosus muscle*; LT, *Longissimus thoracis*.

**Table 3:** Amino acid analysis of muscle from different muscle types of yaks

Items (μg/g)	LT	SM	TB	p-value
Isoleucine (Ile)	16.14±6.44	14.90±3.65	14.44±2.03	0.857
Valine (Val)	34.07±11.54	31.40±6.35	30.69±4.09	0.821
Methionine (Met)	7.10±2.06	6.83±2.02	6.41±0.88	0.854
Leucine (Leu)	23.42±8.35	21.30±4.96	21.97±3.24	0.875
Phenylalanine (Phe)	13.03±6.32	11.08±2.32	10.84±1.59	0.702
Lysine (Lys)	14.70±8.09	13.71±5.22	22.35±10.14	0.291
Threonine (Thr)	14.12±9.75	11.16±2.29	12.49±1.79	0.780
Histidine (His)	711.72±64.56^a^	683.42±58.27^a^	494.61±44.32^b^	<0.001
Arginine (Arg)	14.15±2.75^b^	15.76±3.29^b^	20.31±4.05^a^	0.043
Tyrosine (Tyr)	19.71±3.85^a^	14.25±2.49^b^	13.49±1.24^b^	0.019
Serine (Ser)	49.19±5.10^a^	25.88±3.01^b^	28.48±6.03^b^	0.035
Proline (Pro)	12.73±6.10	12.52±1.36	12.33±2.54	0.989
Glycine (Gly)	72.00±12.58	69.28±18.48	62.96±8.49	0.647
Alanine (Ala)	615.87±32.22	619.68±41.03	600.50±44.74	0.773
Aspartate (Asp)	23.79±20.12	12.58±5.38	19.08±2.75	0.447
Glutamic acid (Glu)	16.02±2.71^b^	13.94±3.88^b^	30.34±10.36^a^	0.008
Asparagine (Asn)	7.09±4.31	5.95±2.16	7.43±3.75	0.826
Ornithine (Orn)	5.72±0.93^b^	4.98±0.28^b^	33.67±13.60^a^	0.017
Homocysteine (Hcy)	0.13±0.03^a^	0.06±0.03^b^	0.03±0.02^b^	0.004
Glutamicaid (Gln)	412.75±99.59^b^	373.08±93.42^b^	967.94±236.63^a^	<0.001
Tryptophan (Trp)	5.02±2.83	4.04±0.78	3.71±0.24	0.545
Total amino acids (TAA)	2,088.76±92.65^b^	1,966.15±74.36^b^	2,414.48±110.63^a^	0.015
Essential amino acids (EAAs)	142.32±32.91	124.68±13.93	132.70±11.10	0.848
Nonessential amino acids (NEAA)	1,949.92±67.46^b^	1,841.54±67.81^b^	2,264.60±100.08^a^	0.007
Flavor amino acid (FAA)	522.70±43.20^b^	470.73±50.44^b^	1,070.31±117.80^a^	<0.001
Sweet amino acid (SAA)	778.63±58.63	752.26±28.34	739.14±26.40	0.785
Bitter amino acid (BAA)	1,223.09±30.15^b^	1,144.71±52.32^b^	1,553.75±104.41^a^	0.003

FAA=(Asp+Glu+Gly+Arg); SAA=(Gly+Ser+Thr+Lys+Pro+Ala); BAA=(His+Met+Val+Arg+Leu+Phe+Trp+Ile+Tyr).

a–c Indicate those with different subscripts in the same row, significant difference at p<0.05.

LT, *Longissimus thoracis*; SM, *semimembranosus muscle*; TB, *triceps brachii*.

**Table 4:** ROAV values of primary flavor compounds in different muscle parts of yak

Compounds	Formula	LT	TB	SM
2-Nonenal, (E)-	C_9_H_16_O	50.62498	51.56003	50.62498
2-Octenal, (E)-	C_10_H_16_O	3.63816	2.821585	3.63816
Heptanal	C_7_H_14_O	23.01183	13.22629	23.01183
Butanal, 2-methyl-	C_5_H_10_O	7.920318	7.753909	7.920318
1-Octen-3-one	C_8_H_14_O	1.067529	0.542431	1.067529
2,3-Butanedione	C_4_H_6_O_2_	100	88.89121	100
Furan, 2-pentyl-	C_9_H_14_O	7.181215	3.766948	7.18
1-Hexanol, 2-ethyl-	C_8_H_18_O	0.949273	0.212046	1.188638

ROAV, relative odor activity value; LT, *Longissimus thoracis*; TB, *triceps brachii*; SM, *semimembranosus muscle*.

## References

[1] Wang G Wang GP Li XP Detection of Giardia duodenalis assemblage E infections at the Tibetan Plateau area: yaks are suitable hosts Acta Trop 2017 169 157 62 10.1016/j.actatropica.2017.02.018 28209552

[2] Guo Z Ge X Yang L Ultrasound-assisted thawing of frozen white yak meat: effects on thawing rate, meat quality, nutrients, and microstructure Ultrason Sonochem 2021 70 105345 10.1016/j.ultsonch.2020.105345 32932225 PMC7786592

[3] Bai X Yin F Ru A Muscle fiber composition affects the postmortem redox characteristics of yak beef Food Chem 2022 397 133797 10.1016/j.foodchem.2022.133797 35917786

[4] Hao L Su W Zhang Y Effects of supplementing with fermented mixed feed on the performance and meat quality in finishing pigs Anim Feed Sci Technol 2020 266 114501 10.1016/j.anifeedsci.2020.114501

[5] Ángel-Rendón SV Filomena-Ambrosio A Hernández-Carrión M Pork meat prepared by different cooking methods. A microstructural, sensorial and physicochemical approach Meat Sci 2020 163 108089 10.1016/j.meatsci.2020.108089 32078892

[6] Kerth CR Miller RK Beef flavor: a review from chemistry to consumer J Sci Food Agric 2015 95 2783 98 10.1002/jsfa.7204 25857365

[7] Zierath JR Hawley JA Skeletal muscle fiber type: influence on contractile and metabolic properties PLOS Biol 2004 2 e348 10.1371/journal.pbio.0020348 15486583 PMC521732

[8] Schiaffino S Reggiani C Fiber types in mammalian skeletal muscles Physiol Rev 2011 91 1447 531 10.1152/physrev.00031.2010 22013216

[9] Song S Ahn CH Song M Pork loin chop quality and muscle fiber characteristics as affected by the direction of cut Foods 2020 10 43 10.3390/foods10010043 33375235 PMC7823467

[10] Wu X Zhou X Chu M Whole transcriptome analyses and comparison reveal the metabolic differences between oxidative and glycolytic skeletal muscles of yak Meat Sci 2022 194 108948 10.1016/j.meatsci.2022.108948 36058093

[11] Kukowski AC Maddock RJ Wulf DM Fausti SW Taylor GL Evaluating consumer acceptability and willingness to pay for various beef chuck muscles J Anim Sci 2005 83 2605 10 10.2527/2005.83112605x 16230658

[12] Tang L Wang Q Xing H Comparative transcriptome analysis reveals the patterns of gene expression in different venison cuts of sika deer (Cervus nippon) Anim Biosci 2025 38 2324 35 10.5713/ab.25.0044 40369752 PMC12580950

[13] Bleicher J Ebner EE Bak KH Formation and analysis of volatile and odor compounds in meat: a review Molecules 2022 27 6703 10.3390/molecules27196703 36235239 PMC9572956

[14] Yang T Wei J Wu X Exploitation of pig by-product: physicochemical properties and flavor of Dachang Tang: effect of cooking time Anim Biosci 2025 38 2266 79 10.5713/ab.24.0894 40302676 PMC12415379

[15] Wang J Liang XF He S Lipid deposition pattern and adaptive strategy in response to dietary fat in Chinese perch (Siniperca chuatsi) Nutr Metab 2018 15 77 10.1186/s12986-018-0315-6 PMC6211486 30410565

[16] Wang Z An X Yang Y Zhang L Jiao T Zhao S Comprehensive analysis of the longissimus dorsi transcriptome and metabolome reveals the regulatory mechanism of different varieties of meat quality J Agric Food Chem 2023 71 1234 45 10.1021/acs.jafc.2c07043 36601774

[17] Want EJ Masson P Michopoulos F Global metabolic profiling of animal and human tissues via UPLC-MS Nat Protoc 2013 8 17 32 10.1038/nprot.2012.135 23222455

[18] Wei W Zha C Jiang A A combined differential proteome and transcriptome profiling of fast- and slow-twitch skeletal muscle in pigs Foods 2022 11 2842 10.3390/foods11182842 36140968 PMC9497725

[19] Renand G Picard B Touraille C Berge P Lepetit J Relationships between muscle characteristics and meat quality traits of young Charolais bulls Meat Sci 2001 59 49 60 10.1016/S0309-1740(01)00051-1 22062505

[20] Bai X Yin F Ru A Myosin heavy chain isoform expression and meat quality characteristics of different muscles in yak (Bos grunniens) Meat Sci 2024 209 109414 10.1016/j.meatsci.2023.109414 38101288

[21] Grimaldi PA Roles of PPARδ in the control of muscle development and metabolism Biochem Soc Trans 2003 31 1130 2 10.1042/bst0311130 14641010

[22] Tokinoya K Ishikura K Yoshida Y LDH isoenzyme 5 is an index of early onset muscle soreness during prolonged running J Sports Med Phys Fitness 2020 60 1020 6 10.23736/s0022-4707.20.10278-0 32253893

[23] Auger C Han S Appanna VP Thomas SC Ulibarri G Appanna VD Metabolic reengineering invoked by microbial systems to decontaminate aluminum: implications for bioremediation technologies Biotechnol Adv 2013 31 266 73 10.1016/j.biotechadv.2012.11.008 23201464

[24] Nagatomo F Fujino H Kondo H The effects of running exercise on oxidative capacity and PGC-1α mRNA levels in the soleus muscle of rats with metabolic syndrome J Physiol Sci 2012 62 105 14 10.1007/s12576-011-0188-1 22234788 PMC10717813

[25] Gao X Wang Z Tang M Ma C Dai R Comparsion of the effects of succinate and NADH on postmortem metmyoglobin redcutase activity and beef colour stability J Integr Agric 2014 13 1817 26 10.1016/s2095-3119(14)60754-1

[26] Cui Y Li J Deng D Solid-state fermentation by Aspergillus niger and Trichoderma koningii improves the quality of tea dregs for use as feed additives PLOS ONE 2021 16 e0260045 10.1371/journal.pone.0260045 PMC8589212 34767609

[27] Zhang W Xiao S Samaraweera H Lee EJ Ahn DU Improving functional value of meat products Meat Sci 2010 86 15 31 10.1016/j.meatsci.2010.04.018 20537806

[28] Hwang YH Ismail I Joo ST Identification of umami taste in sous-vide beef by chemical analyses, equivalent umami concentration, and electronic tongue system Foods 2020 9 251 10.3390/foods9030251 32110974 PMC7143247

[29] Duan Y Zheng C Zheng J Profiles of muscular amino acids, fatty acids, and metabolites in Shaziling pigs of different ages and relation to meat quality Sci China Life Sci 2023 66 1323 39 10.1007/s11427-022-2227-6 36564558

[30] Jiang Y Li D Tu J Mechanisms of change in gel water-holding capacity of myofibrillar proteins affected by lipid oxidation: the role of protein unfolding and cross-linking Food Chem 2021 344 128587 10.1016/j.foodchem.2020.128587 33191014

[31] Fu Y Cao S Yang L Li Z Flavor formation based on lipid in meat and meat products: a review Food Biochem 2022 46 e14439 10.1111/jfbc.14439 36183160

[32] Wood JD Richardson RI Nute GR Effects of fatty acids on meat quality: a review Meat Sci 2004 66 21 32 10.1016/S0309-1740(03)00022-6 22063928

[33] Liu Q Lei M Zhao W Li X Zeng X Bai W Formation of lipid-derived flavors in dry-cured mackerel (Scomberomorus niphonius) via simulation of autoxidation and lipoxygenase-induced fatty acid oxidation Foods 2023 12 2504 10.3390/foods12132504 37444242 PMC10340243

[34] Wood JD Enser M Fisher AV Fat deposition, fatty acid composition and meat quality: a review Meat Sci 2008 78 343 58 10.1016/j.meatsci.2007.07.019 22062452

[35] Voss MD Beha A Tennagels N Gene expression profiling in skeletal muscle of Zucker diabetic fatty rats: implications for a role of stearoyl-CoA desaturase 1 in insulin resistance Diabetologia 2005 48 2622 30 10.1007/s00125-005-0025-2 16284748

[36] Agbaga MP Brush RS Mandal MNA Henry K Elliott MH Anderson RE Role of Stargardt-3 macular dystrophy protein (ELOVL4) in the biosynthesis of very long chain fatty acids Proc Natl Acad Sci USA 2008 105 12843 8 10.1073/pnas.0802607105 PMC2525561 18728184

[37] Hagen RM Rodriguez-Cuenca S Vidal-Puig A An allostatic control of membrane lipid composition by SREBP1 FEBS Lett 2010 584 2689 98 10.1016/j.febslet.2010.04.004 20385130

[38] Elmore JS Cooper SL Enser M Dietary manipulation of fatty acid composition in lamb meat and its effect on the volatile aroma compounds of grilled lamb Meat Sci 2005 69 233 42 10.1016/j.meatsci.2004.07.002 22062813

[39] Glorieux S Steen L De Brabanter J Foubert I Fraeye I Effect of meat type, animal fatty acid composition, and isothermal temperature on the viscoelastic properties of meat batters J Food Sci 2018 83 1596 604 10.1111/1750-3841.14182 29786844

[40] Shi Y Li X Huang A A metabolomics-based approach investigates volatile flavor formation and characteristic compounds of the Dahe black pig dry-cured ham Meat Sci 2019 158 107904 10.1016/j.meatsci.2019.107904 31374425

[41] Gkarane V Brunton NP Harrison SM Volatile profile of grilled lamb as affected by castration and age at slaughter in two breeds J Food Sci 2018 83 2466 77 10.1111/1750-3841.14337 30251256

[42] Yu X Li C Zhao Z Zhang Y Effects of slaughter age on the quality of Gannan yak meat: analysis of edible quality, nutritional value, and GC×GC-ToF-MS of the longissimus dorsi muscle Food Sci Nutr 2025 13 e70381 10.1002/fsn3.70381 40521073 PMC12163752

[43] Wan J Liu Q Ma C Characteristic flavor fingerprint disclosure of dzo beef in Tibet by applying SAFE-GC-O-MS and HS-GC-IMS technology Food Res Int 2023 166 112581 10.1016/j.foodres.2023.112581 36914343

[44] Sun Y Wu Y Liu B Analysis for different flavor compounds in mature milk from human and livestock animals by GC × GC-TOFMS Food Chem X 2023 19 100760 10.1016/j.fochx.2023.100760 37780337 PMC10534127

[45] Yamashita A Hayashi Y Matsumoto N Glycerophosphate/acylglycerophosphate acyltransferases Biology 2014 3 801 30 10.3390/biology3040801 25415055 PMC4280512

[46] Bian Y Wang H Sun S Taurine alleviates endoplasmic reticulum stress in the chondrocytes from patients with osteoarthritis Redox Rep 2018 23 118 24 10.1080/13510002.2018.1445581 29494284 PMC6748701

[47] Watanabe M Houten SM Mataki C Bile acids induce energy expenditure by promoting intracellular thyroid hormone activation Nature 2006 439 484 9 10.1038/nature04330 16400329

[48] Worthmann A John C Rühlemann MC Cold-induced conversion of cholesterol to bile acids in mice shapes the gut microbiome and promotes adaptive thermogenesis Nat Med 2017 23 839 49 10.1038/nm.4357 28604703

[49] Ferrell JM Chiang JYL Understanding bile acid signaling in diabetes: from pathophysiology to therapeutic targets Diabetes Metab J 2019 43 257 72 10.4093/dmj.2019.0043 31210034 PMC6581552

[50] Carillo MR Bertapelle C Scialò F L-carnitine in Drosophila: a review Antioxidants 2020 9 1310 10.3390/antiox9121310 33371457 PMC7767417

[51] Shindou H Hishikawa D Harayama T Eto M Shimizu T Generation of membrane diversity by lysophospholipid acyltransferases J Biochem 2013 154 21 8 10.1093/jb/mvt048 23698096

[52] Li Q Xia Z Wu Y Lysophospholipid acyltransferase-mediated formation of saturated glycerophospholipids maintained cell membrane integrity for hypoxic adaptation FEBS J 2024 291 3191 210 10.1111/febs.17132 38602252

[53] Borrelli GM Troccoli A Fonzo ND Fares C Durum wheat lipoxygenase activity and other quality parameters that affect pasta color Cereal Chem 1999 76 335 40 10.1094/CCHEM.1999.76.3.335

